# Relationship between Physical Activity and Cardiovascular Risk Factors: A Cross-Sectional Study among Low-Income Housewives in Kuala Lumpur

**DOI:** 10.3390/ijerph18116090

**Published:** 2021-06-04

**Authors:** Nur Zakiah Mohd Saat, Siti Aishah Hanawi, Nor M. F. Farah, Hazilah Mohd Amin, Hazlenah Hanafiah, Nur Shazana Shamsulkamar

**Affiliations:** 1 Biomedical Science Programme, Centre of Community Health (ReaCH), Faculty of Health Sciences, Universiti Kebangsaan Malaysia, Kuala Lumpur 50300, Malaysia; shazana2497@gmail.com; 2 SOFTAM, Faculty of Information Science and Technology, Universiti Kebangsaan Malaysia, Bangi 43600, Malaysia; ctaishah@ukm.edu.my (S.A.H.); hazilah@ukm.edu.my (H.M.A.); 3 Occupational Therapy Programme, Centre of Community Health (ReaCH), Faculty of Health Sciences, Universiti Kebangsaan Malaysia, Kuala Lumpur 50300, Malaysia; norfarah@ukm.edu.my; 4 Faculty of Computer and Mathematical Science, Universiti Teknologi MARA Sabah Branch, Kota Kinabalu Campus, Sabah 88997, Malaysia; hazlenahh@uitm.edu.my

**Keywords:** cardiovascular risk factors, physical activity, housewives, obesity and overweight

## Abstract

Cardiovascular disease is a significant public health concern worldwide, including in Malaysia. Various attempts have been made to resolve this issue. One of the most important methods of controlling cardiovascular risk factors is physical exercise. However, today’s women, especially housewives, are often identified by a lack of physical activity. This is alarming to society, as cardiovascular disease can affect the quality of their life. The aim of this study is to determine the relationship between physical activity and cardiovascular risk factors among low-income housewives in Kuala Lumpur. A total of 63 housewives participated in this cross-sectional study. All participating housewives were asked to fill out a sociodemographic questionnaire and the short version of the International Physical Activity Questionnaire (IPAQ). To evaluate cardiovascular risk factors, anthropometric measurements and blood samples were taken. Findings showed that an average of 70.5 ± 232.4 min/week was spent on moderate-to-vigorous physical activity (MVPA), which indicated a low level of physical activity. Data showed that 90.5% of the subjects had low physical activity, 6.3% were moderate, and 3.2% were considered as having a high level of physical activity. For body mass index (BMI), 58.7% of the respondents were obese, 28.6% were overweight (29.10 ± 5.67 kg m^–2^), and 81.0% of subjects had a waist circumference (WC) value above the normal range (92.74 ± 16.40 cm). A two-way ANOVA test revealed significant mean differences between systolic blood pressure (mm/Hg) and age groups (*p* > 0.05). Nevertheless, there was a significant association between MVPA and cardiovascular risk factors using negative binomial regression (*p* < 0.01). The findings of this study highlight the need for health promotional programs to raise awareness, educate, and engage low-income housewives in lifestyle-enhancing behaviors.

## 1. Introduction

Cardiovascular disease (CVD), which is also known as heart disease, is the dominant contributor to death worldwide and a prime contributor in reducing the quality of life of an individual [1]. According to the World Health Organization (WHO, Geneva, Switzerland), cardiovascular disease, especially heart attacks and strokes, were reported in 2016 as major contributors to the increasing mortality rate in the world, where 31% of the global mortality rate was due to this disease [2]. This is also a threat in Malaysia, where the highest percentage of deaths in Malaysia was recorded due to cardiovascular disease, at 35% [3]. 

Several causes, including metabolic factors such as hyperglycemia, hypertension, obese or overweight, and hypercholesterolemia, all contribute to the progression of this cardiovascular disease. Furthermore, environmental factors (lack of physical exercise, heavy alcohol consumption, smoking habits, and an unhealthy diet) contribute to the disease prevalence [4,5]. As a result, several public health authorities are focusing on these risk factors to combat the burden of cardiovascular disease, as they have been shown to lower the death rate of noncommunicable diseases, especially cardiovascular disease [6]. 

Consistent physical exercise has several advantages, especially in terms of recent and long-term health. Physical exercise will minimize the risk of premature death, heart disease, metabolic syndrome, cancer, and depression and improve sleep quality, according to the Ministry of Health Malaysia’s recommendations [7]. Regular physical exercise, as well as physical health, can influence blood sugar levels, blood pressure levels, weight, and lipid levels in the body [8,9]. Individuals who participate in regular walking routines and strength training effectively lowered their waist circumference dimensions and overall body weight [10]. 

Nonetheless, many women today, especially housewives and retirees, lead sedentary lifestyles that lead to physical inactivity [11,12,13]. Housewives’ duties have been said to discourage them from participating in prescribed physical activity, as they are busy managing the household and taking care of the children [14]. This puts this demographic segment at a greater risk of developing a condition as a result of long-term physical inactivity, such as cardiovascular disease.

Due to their hectic schedules and lack of physical exercise, those who live in metropolitan areas and have a low income are at a higher risk of acquiring cardiovascular illnesses [4]. Moderate physical exercise can help to minimize or eliminate the chance of acquiring the condition [10]. The Ministry of Health Malaysia recommends that individuals aged from 16 to 64 engage in moderate physical activity for 150 min per week or 75 min per week at high intensity. However, housewives often do not have time to engage in the recommended amount of physical exercise [6]. Because there is a lack of information on housewives with low income in Malaysia, this study is important to provide additional information about this group.

Previous research has concentrated on blue-collar employees, office workers, students, and teachers, but there has been little research on housewives, especially in Malaysia. Blue-collar employees [15], office workers [16], and students [17] have been shown to be more likely to exhibit cardiovascular risk factors due to unhealthy lifestyles such as lack of physical activity and unhealthy diet, while teachers have a better lifestyle and therefore showing lesser cardiovascular risk factors [18]. Therefore, the aim of this study was to assess if there is a relationship between physical activity and cardiovascular risk factors among housewives in the low-cost housing provision in Kuala Lumpur.

## 2. Materials and Methods

This cross-sectional study was conducted in the low-cost housing provision located in metropolitan Kuala Lumpur. The subjects were housewives who met the inclusion criteria and voluntarily participated. Subjects who were disabled or had a chronic disease, pregnant, and had a history of cardiovascular disease were excluded from this study.

This study was approved by the Universiti Kebangsaan Malaysia Research Ethics Committee. A self-administered and guided questionnaire consisting of the sociodemographic background of age, duration of being a housewife, number of children, education level, and household income, as well as the International Physical Activity Questionnaire (IPAQ)-short form, was distributed to the suitable housewives. Subjects who agreed to participate in the study signed a form confirming informed consent.

Physical activity was assessed by using the International Physical Activity Questionnaire (IPAQ)-short form through an interview session. The questionnaire was tested and validated for its reliability among the Malay population in Malaysia by another researcher [19]. This component consists of 7 questions regarding the physical activity performed during the last 7 days, which include vigorous-intensity and moderate-intensity physical activity, walking, and sitting. Details on the length of time spent performing physical activities on both weekdays and weekends were acquired during the interview session. Physical activity was scored by using the following formula:

MET-min/week = min of activity/day × day per week × MET level

The level of physical activity was classified into three categories, which are low (<600 MET-minutes/week), moderate (600–3000 MET-minutes/week), and high (≥3000 MET-minutes/week) according to the guidelines of the IPAQ [20]. In order to determine moderate-to-vigorous physical activity, the total score of the IPAQ was applied, using the categories sedentary (≤100 counts/min), light (101–1951 counts/min), moderate (1952–5724 counts/min), and vigorous (≥5725 counts/min) [21].

Biomarkers of cardiovascular risk comprised indicators for overweight and obesity (body mass index, waist circumference), blood pressure, fasting blood glucose, and total cholesterol. Weight was measured using an analog scale to the nearest 0.1 kg, without shoes, with light clothing, and items removed from the subjects’ pockets before being weighed. Height was measured to the nearest 0.5 cm by using a portable height measuring device (Seca 213). Body mass index (BMI) was calculated as weight in kilograms divided by height in meters squared (kg m^–2^) and categorized according to cut-off points for Asian population, which are: which are underweight (<18.5), normal (18.5–22.9), overweight (23.0–27.49), and obesity (≥27.5). Waist circumference (WC), an indicator of abdominal obesity, was taken at the midpoint between the top of the iliac crest and the lower margin of the last palpable midaxillary line at minimal respiration of the normal expiration. The data were analyzed by using cut-off points for Asian population (≥80 cm for women) [22]. Blood pressure (BP) was measured by using a digital sphygmomanometer (Omron Inc., Osaka, Japan) with a suitable cuff size with the subject seated and rested. Hypertension was defined as systolic blood pressure (SBP) of ≥140 mmHg, diastolic blood pressure (DBP) of ≥90 mmHg, or a combination of both. Fasting blood glucose (FBG) and total cholesterol (TC) were measured using finger-prick samples on portable meters. An FBG value between 3.9 and 5.5 mmol/L^–1^ was considered normal, as was a TC of ≤5.2 mmol/L^–1^ [23,24].

The sample size that was required for this study was 68 according to formulae by Cochran [25]: *n* = (z_¦Á/2_)^2^ *p*(1-*p*)/∆^2^; *p* = prevalence of diabetes, 17.5%, according to the National Health Morbidity Survey in Malaysia (NHMS) [24]; z = 1.96;Δ = margin of error, 9%. The inclusion criteria for this study were: (i) participant was a housewife with no permanent income from a job; (ii) participant was a housewife for at least 2 years. Data were analyzed by using the Statistical Package for the Social Sciences (IBM SPSS Statistics Version 23.0). All data were tested for normality. Two-way ANOVA, one-way ANOVA, the Mann–Whitney U test, and the Kruskal–Wallis H test were applied to test the significant mean differences between groups. The relationship between the response variable MVPA and the predictor variable was determined by using negative binomial regression.

## 3. Results

Table 1 shows the sociodemographic characteristics of the 63 subjects who participated in this study. The subjects were mostly aged between 51 and 60 years (41.3%) and had been a housewife for over 20 years (44.4%). The majority of the respondents had three to four children (47.6%), did not possess a tertiary education, and had an estimated household income in the range of RM 1001 to RM 2000 (46.0%).

Table 2 presents the distribution of cardiovascular risk factors for low-income housewives in Kuala Lumpur. For BMI, 58.7% of housewives were indicated as obese, and 28.6% were overweight (29.10 ± 5.67). About 81.0% of housewives were obese, as the WC exceeded the normal range of 80 cm, with an overall mean of 92.74 ± 16.40. Most housewives had normal BP, where 52.4% showed normal systolic blood pressure, and 33.3% were prehypertensive, with an overall mean of 122.90 ± 19.05, which indicates they were prehypertensive. Similarly, 50.8% had normal diastolic blood pressure, and 28.6% showed prehypertension, with an overall mean indicating prehypertension of 81.04 ± 10.43. A total of 49.2% of housewives had normal FBG levels, and 31.7% were at prediabetic levels (6.48 ± 2.81). As for TC, a total of 92.1% of housewives had normal cholesterol levels, and 6.3% were at high border levels (4.15 ± 0.72).

Table 3 demonstrates the results of the mean difference between CVD risk factors, age group, and education level using two-way ANOVA. The data showed that there was no significant mean difference between BMI, WC, DBP, FBG, and TC with age group as the main factor. Despite that, SBP (F = 3.49, *p* < 0.05) showed significant mean differences with age group. A post-hoc test was performed on these significant parameters and revealed that there is a significant mean difference between the age group of 21–40 years and the age group of 51–60 years (mean difference = −17.59, *p* = 0.006). Furthermore, there was no significant mean difference in BMI, WC, SBP, DBP, FBG, and TC with education level as the main factor (*p* > 0.05). 

Furthermore, there were no significant mean differences between the CVD risk factor and family income in Table 4 (*p* > 0.05).

According to Table 5, a total of 57 of the housewives had low physical activity, which accounted for 90.5%. About four housewives showed moderate physical activity (6.3%), followed by two housewives who had a high level of physical activity (3.2%). The overall mean of total physical activity obtained from this study is 451.35 ± 617.78 MET-min/week, which indicates that the level of physical activity among housewives is low. For the mean of moderate-to-vigorous physical activity (MVPA), the data showed that housewives were physically inactive (70.48 ± 282.36 min/week) because they did not reach 150 min/week as recommended. The average sitting time of a housewife is 2.92 ± 1.49 h a day.

**Figure 1:**
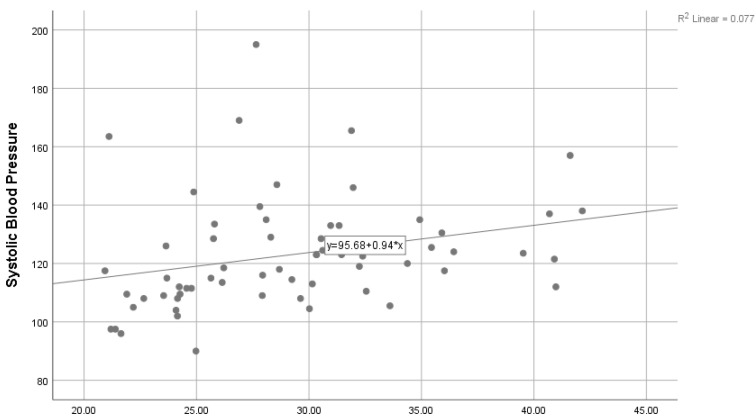
Correlation between SBP (mm/Hg) and BMI (kg/m^2^).

**Figure 2:**
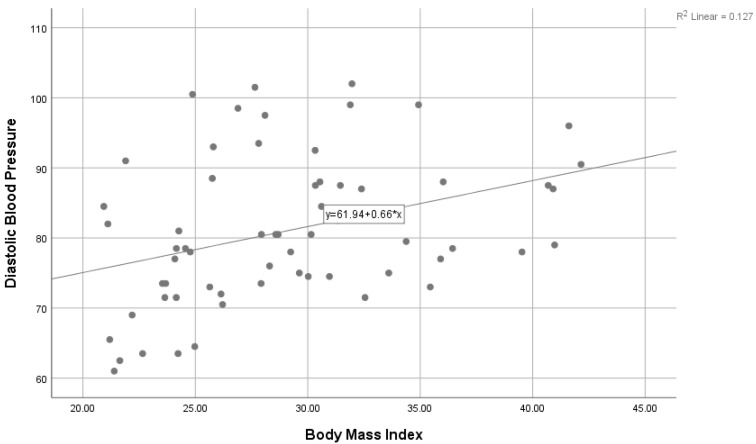
Correlation between DBP (mm/Hg) and BMI (kg/m^2^).

Moreover, Table 6 presents the mean differences between physical activity level, age group, education level, and household income. Based on these analyses, the results indicate that there were no significant median differences between physical activity level; age group, H(2) = 4.70; education level, U = 393; and household income, H(2) = 3.62 (*p* > 0.05). Figure 1 shows a scatter plot between SBP (mm/Hg) and BMI (kg/m^2^). There was a significant positive correlation with the correlation coefficient r = 0.284 (*p* < 0.05). Furthermore, there was a significant positive correlation between DBP (mm/Hg) and BMI (kg/m^2^), with r = 0.784 and *p* < 0.001, as shown in Figure 2.

According to Table 7, negative binomial regression (NBR) was chosen because of the overdispersion in the data of MVPA. The standard deviation of MVPA was higher compared to the mean of MVPA in this study. Based on the likelihood ratio, Pearson’s chi-squared test provided the overall model compared to the model without any predictors, showing that this model was significant (χ^2^ = 90.06, *p* < 0.001). Table 7 further presents the NBR coefficient for each predictor and standard errors. The variable FBG has a coefficient of −0.570 (*p* < 0.001). This indicates for each one-unit increase in FBG, the expected log count of MVPA decreases by −0.57. Furthermore, the variable TC has a coefficient of −3.687 (*p* < 0.01). This indicates for each one-unit increase in TC, the MVPA decreases by −3.687 units. SBP and DBP were also statistically significant, with coefficients of 0.09 and −0.268, respectively (*p* < 0.001).

## 4. Discussion

According to the findings of this study, housewives are more likely to be overweight or obese, which puts them at risk of cardiovascular disease. The incidence of overweight and obesity was found to be higher in this sample than in the NHMS 2015 (Malaysia [24]) and the World Health Organization [26], with prevalence rates of 54.4% and 52.0%, respectively. The percentage of obesity in this study is parallel with the findings of NHMS 2015. Furthermore, the prevalence of obesity among women in Malaysia was somewhat higher than in Spain when comparing the NHMS population study (54.4%) in 2015 with the Spanish population research in 2015 (33.6%) [24,27]. The age range for adults in this research was 21 to 60, whereas the age range for NHMS 2019 was 18 to 64 [23]. As a result, there were minor differences in age groupings, which may have influenced the prevalence results. In addition, compared to other countries such as Spain (33%) [27] and Indonesia (28%), the respondents of this study recorded a high prevalence of obesity in the abdominal region, also known as abdominal obesity, which is measured by waist circumference [28]. As a result, more proactive advertising to raise awareness, particularly among Malaysian women, is needed to address these issues [15,23].

As a result, BMI and blood pressure rise in parallel with age, raising the risk of cardiovascular disease. Another study found that BMI, waist circumference, blood pressure, fasting glucose levels, triglycerides, low-density lipoprotein (LDL), and the ratio of total cholesterol to high-density lipoprotein (HDL) increase with age [29]. Similarly, a study in Kathmandu, Nepal, discovered that the prevalence of overweight or obese, as well as the prevalence of hypertension, increased with age until the age of 50 to 59 years [11]. These results are also consistent with the findings of the authors of [30], who found that people in their 50s and 60s had the greatest incidence of hypertension. This suggests a rise in cardiovascular risk as a result of age-related causes.

Low socioeconomic status, which includes schooling, income, and jobs, is often linked to poor health [4,31,32,33]. Adults in Sabah with lower levels of education are more likely to have hypertension and hypercholesterolemia, according to a study in 2019 [31]. Additionally, people who have a lower household income and have never been diagnosed with noncommunicable disorders have slightly higher systolic blood pressure, overall cholesterol, LDL cholesterol, and fasting blood glucose. This may be attributed to a lack of understanding about the benefits of routine check-ups. This is reinforced by the finding that women with a lower education level have a slightly higher incidence of cardiovascular risk factors [4]. This is due to a lack of accessibility and comprehension of health statistics. Women with a lower education level are often more likely to be ignorant of healthy lifestyle habits. Analysis of 882 Malaysians found that people with no educational qualifications and an income of less than RM 1000 are more likely to have a higher likehood of cardiovascular incidence in the next ten years [32]. However, the data of this study found that there is no significant difference between the factors of individual education level and household income with cardiovascular risk factors.

The majority of the participants in this sample had low levels of physical exercise, supplemented by moderate and high levels. This study’s low prevalence of physical activity is higher than those seen in Somaliland (78.4%) [29], Kathmandu (61.1%) [11], Bolivia (64.77%) [33], and Iran (52.2%) [34]. Previous Malaysian surveys, on the other hand, have found reverse findings, with the bulk of respondents (81.9%) being physically involved. In a previous study, there was a significant association between physical activity level and BMI [35]. In this paper, housewives spent the bulk of their time doing moderate-intensity physical exercise, averaging 70.48 ± 282.36 MET-min a week. This illustrates that their amount of physical exercise is inadequate, as it falls short of the 150 min a week recommended by the Malaysian Ministry of Health Malaysia [6] and the World Health Organization (WHO) [3]. This may be attributed to a lack of time for housewives to engage in physical activity due to their responsibilities as housewives, as well as a lack of desire and motivation.

Furthermore, the results of this research indicate that there is no major association between physical activity levels and age, schooling, or income. Physical activity levels in the high-income community and highly educated individuals are observed to decline with rising age [12,36,37]. Bad health, such as heart, knee or back issues, arthritis, lack of a companion to perform physical exercise with, lack of interest in recreation, fear of falling, and pain, are typical obstacles to physical activity in older adults, according to previous studies [38,39]. Furthermore, people with lower levels of education and income are more likely to work in physically demanding occupations, resulting in higher levels of physical activity [40]. However, there is a connection between elevated levels of physical activity and high socioeconomic status in developed countries [41,42]. Individuals with a higher socioeconomic status have a greater awareness of the health benefits of physical exercise, especially recreational activities, and can therefore participate in more physical activity even when they are not employed [40].

In this research, there was only a significant relationship between physical exercise and cardiovascular risk. According to a previous study, some metabolic risk factors and physical activity groups have a statistically important negative association [43]. Furthermore, there is no connection between inactivity and overweight or obese in Malaysian men and women [9,44,45]. Except for triglyceride levels, a previous study observed little difference in the impact of physical exercise on glucose levels and lipid profile. In contrast, other studies in Malaysia found no significant relationship between physical exercise and cardiovascular risk factors in their studies [14,46]. This may be because other considerations such as calorie consumption, number of cigarettes smoked a day, and chronic stress are not taken into account [47]. Nonetheless, another study found a strong negative relationship between physical activity and overall cholesterol and total triglycerides, which is consistent with the results of this study [48]. Cardiorespiratory exercise exercises have a clear inverse association with metabolic syndrome, with waist circumference becoming the most significant factor [49].

There are several limitations in this study, such as the small sample size and the location of the study, which was limited to the Kuala Lumpur area. Therefore, the findings cannot be generalized beyond this particular group. Additionally, the study was conducted among housewives living in high-rise residential buildings, which also may have influenced their levels of physical activity, as opposed to living in ground-level housing. With regard to measurement of physical activity levels using self-reported methods, we do not deny that there is a tendency for subjects to overestimate their physical activity, especially when it comes to reporting moderate-to-vigorous physical activity. Thus, we highly recommend the usage of objective measurements of physical activity such as pedometry or accelerometry in future studies. In addition, this study also lacks the measurement of LDL and HDL cholesterol, as well as triglycerides as cardiovascular risk markers. However, as a majority of the sample showed normal cholesterol levels, it can be reasonable to assume that the LDL levels may well be within normal limits. Nevertheless, information on HDL may prove to be useful for future studies to observe how physical activity levels may affect HDL levels in this particular group.

## 5. Conclusions

In conclusion, the majority of housewives in this study are physically inactive and are at risk of cardiovascular disease, as the majority of the sample is obese or overweight. Furthermore, low levels of MVPA are associated with increased cardiovascular risk factors among low-income housewives in Kuala Lumpur. Lifestyle-enhancing intervention programs may serve as a functional avenue to motivate low-income housewives to engage in physical activity and healthy behaviors to improve their cardiovascular risk factors and, ultimately, their quality of life.

## Figures and Tables

**Table 1:** Sociodemographic characteristic of the housewives (*n* = 63).

Characteristics	*n* (%)
**Age**	
21–30	4 (6.3)
31–40	14 (22.2)
41–50	19 (30.2)
51–60	26 (41.3)
**Duration of Being a Housewife (years)**	
≤10	26 (41.3)
11–20	9 (14.3)
>20	28 (44.4)
**Number of Children**	
≤2	10 (15.9)
3–4	30 (47.6)
≥5	23 (36.5)
**Education Level**	
Primary	9 (14.3)
Lower secondary	11 (17.5)
Upper secondary	41 (65.1)
Certificate and above	2 (3.1)
**Household Income**	
≤RM 1000	10 (15.9)
RM 1001–RM 2000	29 (46.0)
>RM 2000	24 (38.1)

**Table 2:** Distribution of CVD risk factors for housewives (*n* = 63).

CVD Risk Factors	*n* (%)	Mean ± SD
**BMI (kg m^−2^)**		
Underweight	0 (0)	29.10 ± 5.67
Normal	8 (12.7)	
Overweight	18 (28.6)	
Obesity	37 (58.7)	
**WC (cm)**		
Normal (≤80 cm)	12 (19.0)	92.74 ± 16.40
Obesity (>80 cm)	51 (81.0)	
**Systolic Blood Pressure (mm Hg)**		
Normal	33 (52.4)	122.90 ± 19.05
Prehypertension	21 (33.3)	
Hypertension stage I	5 (7.9)	
Hypertension stage II	4 (6.4)	
**Diastolic Blood Pressure (mm Hg)**		
Normal	32 (50.8)	81.04 ± 10.43
Prehypertension	18 (28.6)	
Hypertension stage I	10 (15.9)	
Hypertension stage II	3 (4.7)	
**FBG level (mmol L^−1^)**		
Normal	31 (49.2)	6.48 ± 2.81
Prediabetes	20 (31.7)	
Diabetes	12 (19.1)	
**TC Level (mmol L^−1^)**		
Normal	58 (92.1)	4.15 ± 0.72
Borderline high	4 (6.3)	
High	1 (1.6)	

**Table 3:** Mean and standard deviation differences between CVD risk factors, age group, and education level.

Age Group A	Mean ± SD		
	21–40 (*n* = 18)	41–50 (*n* = 19)	51–60 (*n* = 26)
BMI (kg m^−2^)	28.28 ± 6.37	30.30 ± 5.89	28.79 ± 5.13
WC (cm)	88.17 ± 11.29	95.35 ± 23.66	93.98 ± 10.68
SBP (mm Hg)	112.14 ± 11.29	123.74 ± 14.99	129.73 ± 22.78
DBP (mm Hg)	76.19 ± 9.14	84.24 ± 10.43	82.06 ± 10.44
FBG level (mmol L^−1^)	5.26 ± 0.61	7.00 ± 3.08	6.93 ± 3.32
TC level (mmol L^−1^)	3.98 ± 0.59	4.07 ± 0.64	4.33 ± 0.82
**Education Level**	**Mean ± SD**		
	**Primary and Lower Secondary** **(*n* = 20)**	**Upper Secondary and Above** **(*n* = 43)**	
BMI (kg m^−2^)	28.09 ± 5.30	29.57 ± 5.83	
WC (cm)	92.63 ± 22.42	92.78 ± 13.02	
SBP (mm Hg)	122.05 ± 18.62	123.29 ± 19.45	
DBP (mm Hg)	79.30 ± 8.66	81.85 ± 11.16	
FBG level (mmol L^−1^)	6.32 ± 2.47	6.54 ± 2.97	
TC level (mmol L^−1^)	4.19 ± 0.99	4.13 ± 0.55	

**Table 4:** Mean differences between CVD risk factors and household income.

CVD Risk Factor	Mean ± SD				
	≤RM 1000 (*n* = 10)	RM 1001–RM 2000 (*n* = 29)	>RM 2000 (*n* = 24)	F	*p*-Value
BMI (kg m^−2^)	26.25 ± 5.83	29.59 ± 5.55	29.71 ± 5.63	1.54	0.22
WC (cm)	86.24 ± 8.20	92.55 ± 12.46	95.67 ± 21.96	1.18	0.32
SBP (mm Hg)	120.35 ± 17.23	123.38 ± 16.38	123.38 ± 23.06	0.10	0.90
DBP (mm Hg)	77.20 ± 8.35	83.24 ± 10.30	79.98 ± 11.09	1.47	0.24
FBG level (mmol L^−1^)	7.16 ± 4.63	6.50 ± 2.67	6.16 ± 1.97	0.44	0.67
TC level (mmol L^−1^)	4.02 ± 0.83	4.04 ± 0.54	4.34 ± 0.84	1.35	0.27

**Table 5:** Status of physical activity based on the IPAQ (*n* = 63).

Parameter	*n* (%)	Mean ± SD
**Category of Physical Activity Level**		
Low	57 (90.5)	-
Moderate	4 (6.3)	-
High	2 (3.2)	-
**Physical Activity Level**		
Total physical activity level (MET-min/week)	-	451.35 ± 617.78
MVPA (MET-min/week)	-	70.48 ± 282.36
Sitting (h/day)	-	2.92 ± 1.49

**Table 6:** Mean differences between physical activity level, age group, education level, and household income.

Factors	Physical Activity Level					
	*n*	Mean	Median	IQR	H	*p*-Value
**Age**						
21–40	18	452	347	260		
41–50	19	604.05	297	396		
>50	26	321.35	198	355	4.70 ^a^	>0.05
**Education Level**						
Primary and lower secondary	20	294.5	264	247		
Upper secondary and others	43	513.56	240	445	393.00 ^b^	>0.05
**Household Income**						
≤RM 1000	10	250.90	214.50	334		
RM 1001–2000	29	352.14	198.00	256		
>RM 2000	24	635.50	396.00	656	3.62 ^a^	>0.05

a: Kruskal–Wallis test, b: Mann–Whitney U test.

**Table 7:** Negative binomial regression between MVPA and predictors.

Parameter	Coefficients	Std Error	95% Confidence Interval		*p*-Value
Intercept	30.902	4.3320	22.412	39.393	0.000
WC	0.050	0.0737	−0.094	0.195	0.495
BMI	−0.092	0.1625	−0.410	0.226	0.571
FBG	−0.570	0.1360	−0.836	−0.303	<0001 *
TC	−3.687	0.5994	−4.862	−2.512	<0001 *
SBP	0.090	0.0294	0.033	0.148	0.002
DBP	−0.268	0.0489	22.412	39.393	<0001

* *p* < 0.001.

## Data Availability

The corresponding author will provide the data used in this analysis upon request.
